# Supplementary material for: Exponentially-enhanced quantum sensing with non-Hermitian lattice dynamics
Source: Nat Commun. 2020 Oct 23;11:5382. doi: 10.1038/s41467-020-19090-4 (PMC7585448; doi:10.1038/s41467-020-19090-4)
Supplement: Supplementary file 1 — Supplementary Information [file 41467_2020_19090_MOESM1_ESM.pdf]

Supplementary information for: Exponentially enhanced quantum sensing with non-Hermitian lattice dynamics

McDonald and Clerk

Supplementary Note 1. Total Photon Number

Let us compute the total steady-state intracavity photon number on each site to zeoreth order in ϵ. To do so, we must solve the Heisenberg-Langevin equations for the cavity annihilation operators $\hat{a}_n$. Recall that we were able to define new squeezed annihilation and creation operators

$$\hat{a}_n = \cosh(A(n-1))\hat{\tilde{a}}_n + \sinh(A(n-1))\hat{\tilde{a}}_n^\dagger \tag{1}$$

where the Hamiltonian $\hat{H}_B$ conserved the total number of quasiparticles. Thus, the total number of photons on site n reads

$$\begin{aligned}\langle \hat{a}_n^\dagger \hat{a}_n\rangle &= \cosh(2A(n-1))\langle \hat{\tilde{a}}_n^\dagger \hat{\tilde{a}}_n\rangle \\ &+ \sinh(2A(n-1))\,\mathrm{Re}(\langle \hat{\tilde{a}}_n \hat{\tilde{a}}_n\rangle) \\ &+ \sinh^2(A(n-1))\end{aligned} \tag{2}$$

The last term is due to noise that enter the port on site 1 and is turned into real photons by the parametric amplifier-type interactions. We can readily solve the Heisenberg-Langevin equations for the squeezed modes $\hat{\tilde{a}}_n$:

$$\begin{aligned}\hat{\tilde{a}}_n(t) &= \tilde{\chi}(n,m;t)\hat{\tilde{a}}_m(t) \\ &- \sqrt{\kappa}\beta \int_0^t dt' \tilde{\chi}[n,1;t-t'] \\ &- \sqrt{\kappa} \int_0^t dt' \tilde{\chi}[n,1;t-t']\hat{a}^{(\mathrm{in})}(t')\end{aligned} \tag{3}$$

where $\hat{a}^{(\mathrm{in})}(t) = (\hat{X}^{(\mathrm{in})}(t) + i\hat{P}^{(\mathrm{in})}(t))/\sqrt{2}$ is the operator equivalent of Gaussian white noise. Note that we're using Einstein summation notation. Assuming a zero temperature environment we have in the steady-state

$$\langle \hat{\tilde{a}}_n^\dagger \hat{\tilde{a}}_n\rangle = \langle \hat{\tilde{a}}_n \hat{\tilde{a}}_n\rangle = \kappa\beta^2 |\tilde{\chi}[n,1;\omega=0]|^2. \tag{4}$$

Using the relevant susceptibilities computed in Methods, we then obtain

$$\begin{aligned}\langle \hat{a}_n^\dagger \hat{a}_n\rangle = \frac{4\beta^2}{\kappa} e^{2A(n-1)} \sin^2 \frac{\pi}{2} n \\ + \sinh^2(A(n-1))\end{aligned} \tag{5}$$

where we have assumed (and will do so throughout) that N is odd. Summing Eq. (5) over all lattice sites gives

$$\begin{aligned}\bar{n}_{\mathrm{tot}} &= \frac{4|\beta|^2}{\kappa} \frac{e^{2A(N+1)} - 1}{e^{4A} - 1} \\ &+ \frac{1}{4}\left(\frac{\sinh(A(2N-1))}{\sinh(A)} - (2N-1)\right) \\ &= \bar{n}_N \frac{1 - e^{-2A(N+1)}}{1 - e^{-4A}} + \bar{n}_{\mathrm{vac}}\end{aligned} \tag{6}$$

with $\bar{n}_{\mathrm{vac}}$ the photons that are present due to amplified vacuum fluctuations. Thus, the ratio of the average photon number on the last site to the total number of photons $Z(A)$ is

$$Z(A) = \left(\frac{1 - e^{-2A(N+1)}}{1 - e^{-4A}} + \frac{\bar{n}_{\mathrm{vac}}}{\bar{n}_N}\right)^{-1} \tag{7}$$

In the limit where $|\beta|^2/\kappa$ is large, the coherent photons dominate $\bar{n}_{\mathrm{vac}}$, which we can ignore. We then have

$$Z(A) = \frac{1 - e^{-4A}}{1 - e^{-2A(N+1)}} = 1 - \mathcal{O}(e^{-4A}) \tag{8}$$

as in the main text.

Supplementary Note 2. Single-Pole Approximation

As mentioned in the main text, we need to understand finite-time dynamics of our non-Hermitian lattice sensor. While we have the exact frequency-space susceptibilities (computed in Methods) to zeroth-order in ϵ, Fourier transforming to the

time-domain becomes an intractable problem. Note that this is only true of the signal: to zeroeth order in ϵ, the noise is always vacuum.

There is however an exact form of the SNR in the limit where the hopping is infinite $J \to \infty$. In this limit the susceptibilities take the form

$$\tilde{\chi}[N,1;\omega] = \frac{2i^N}{N+1}\frac{1}{\omega + i\frac{\kappa}{N+1}} \tag{9}$$

$$\tilde{\chi}[1,N;\omega] = \frac{-2i^{-N}}{N+1}\frac{1}{\omega + i\frac{\kappa}{N+1}} \tag{10}$$

such that the width of the zero mode is $\kappa/(N+1)$. The Fourier transform of each susceptibility (and their product, which is what determines linear response) is then easily computed.

The change to the cavity quadrature amplitude $\hat{p}_1$ at a time t in response to the perturbation ϵ can be found using Eq. (3) and first order perturbation theory

$$\begin{aligned}\langle \hat{p}_1(t)\rangle &= -\sqrt{2\kappa}\beta \int_0^t dT \delta\chi^{px}(1,1;T) \\ &= \sqrt{2\kappa}\epsilon\beta \int_0^t dT \int_0^T dT' \chi^{pp}(1,N;T-T')\chi^{xx}(N,1;T')\end{aligned} \tag{11}$$

Using $\chi^{xx}(n,m;t) = e^{A(n-m)}\tilde{\chi}(n,m;t)$, $\chi^{pp}(n,m;t) = e^{-A(n-m)}\tilde{\chi}(n,m;t)$, Eqs. (9) and (10) we get

$$\langle \hat{p}_1(t)\rangle = -\epsilon\sqrt{2\kappa}\beta(\frac{2}{N+1})^2 e^{2A(N-1)} \int_0^t dT T e^{-\frac{\kappa T}{N+1}} \tag{12}$$

From which we obtain the signal

$$\begin{aligned}&\mathcal{S}_\tau(N,\epsilon,J\to\infty) = \\ &\frac{\sqrt{2}\kappa|\beta||\epsilon|}{\sqrt{\tau}}(\frac{2}{N+1})^2 e^{2A(N-1)} \int_0^\tau dt \int_0^t dT T e^{-\frac{\kappa T}{N+1}}\end{aligned} \tag{13}$$

whereas the noise is always just $\mathcal{N}_\tau(N,\epsilon) = 1/\sqrt{2}$. The SNR for finite τ is then

$$\begin{aligned}&\mathrm{SNR}_\tau(N,\epsilon,J\to\infty) = \\ &\sqrt{\frac{\tau}{\tau_M^*(N)}}\left(1 + e^{-\frac{\tau}{t_{esc}(N)}} - \frac{2t_{esc}(N)}{\tau}(1 - e^{-\frac{\tau}{t_{esc}(N)}})\right)\end{aligned} \tag{14}$$

where recall

$$\tau_M^*(N) = \frac{1}{16Z(A)\bar{n}_{\mathrm{tot}}\kappa}\left(\frac{\kappa}{\epsilon_0}\right)^2 e^{-2A(N-1)} \tag{15}$$

is the measurement time when the steady-state expression holds.

We now want to find the measurement time $\tau_M^{J=\infty}(N)$ where in both the weak and strong measurement limit. In the weak measurement limit $\tau_M^*(N) \gg t_{esc}(N)$, we recover the steady state result $\tau_M^{J=\infty}(N) = \tau_M^*(N)$. In the strong measurement limit $\tau_M^*(N) \ll t_{esc}(N)$, we seek the leading order contribution to the measurement time. To that end, let us define

$$\gamma = \frac{\tau_M^{J=\infty}(N)}{\tau_M^*(N)} \tag{16}$$

so that

$$\left[\sqrt{\gamma}\left(1 + e^{-\gamma\frac{\tau_M^*(N)}{t_{esc}(N)}}\right) - \frac{2t_{esc}(N)}{\tau_M^*(N)\sqrt{\gamma}}\left(1 - e^{-\gamma\frac{\tau_M^*(N)}{t_{esc}(N)}}\right)\right]^2 = 1 \tag{17}$$

Assuming that $\gamma\tau_M^*(N)/t_{esc}(N)$ is small (which can be verified to be self-consistent after solving for γ), then we can Taylor expand the exponential to third order and obtain

$$\gamma^5 = \left(\frac{\sqrt{6}t_{esc}(N)}{\tau_M^*(N)}\right)^4 \tag{18}$$

from which

$$\tau_M^{J=\infty}(N) = \sqrt{6}t_{esc}(N)\sqrt[5]{\frac{\tau_M^*(N)}{\sqrt{6}t_{esc}(N)}} \tag{19}$$

as in the main text.

Supplementary Note 3. Non-perturbative effects of ϵ_0: wavefunctions, spectrum and output field.

We now want to consider the full effect of ϵ_0 on the wavefunction, spectrum and the output field. To do so, we must compute the susceptibilities $\chi^{\alpha\beta}_{\epsilon_0}[1,1;\omega]$ to all orders in ϵ_0. The full Heisenberg-Langevin equations are

$$\dot{\hat{x}}_n = -i[\hat{x}_n, \hat{H}_B + \epsilon_0 \hat{a}^\dagger_N \hat{a}_N] - \delta_{n1}\left(\frac{\kappa}{2}\hat{x}_n + \sqrt{\kappa}\hat{X}^{(\text{in})}\right) \tag{20}$$

$$\dot{\hat{p}}_n = -i[\hat{p}_n, \hat{H}_B + \epsilon_0 \hat{a}^\dagger_N \hat{a}_N] - \delta_{n1}\left(\frac{\kappa}{2}\hat{p}_n + \sqrt{\kappa}\hat{P}^{(\text{in})}\right), \tag{21}$$

where as in the main text we have incorporated the drive tone amplitude in the definition of the input operators $\langle \hat{X}^{(\text{in})}\rangle = \beta$ and $\langle \hat{P}^{(\text{in})}\rangle = 0$.

Our strategy for solving the Heisenberg-Langevin equations will be nearly identical to that presented when $\epsilon_0 = 0$. The key difference is that our squeezing transformation is now defined as

$$\hat{x}_n = e^{A(n-N)}\hat{\tilde{x}}_n \tag{22}$$

$$\hat{p}_n = e^{-A(n-N)}\hat{\tilde{p}}_n. \tag{23}$$

In this new frame, the Hamiltonian $\hat{H}_B + \epsilon_0 \hat{\tilde{a}}^\dagger_N \hat{\tilde{a}}_N$ preserves total quasiparticle number $\hat{\tilde{N}}$. The Heisenberg-Langevin equations are then

$$\dot{\hat{\tilde{x}}}_n = -i[\hat{\tilde{x}}_n, \hat{H}_B + \epsilon_0 \hat{\tilde{a}}^\dagger_N \hat{\tilde{a}}_N] - \delta_{n1}\left(\frac{\kappa}{2}\hat{\tilde{x}}_n + e^{A(N-1)}\sqrt{\kappa}\hat{X}^{(\text{in})}\right) \tag{24}$$

$$\dot{\hat{\tilde{p}}}_n = -i[\hat{\tilde{p}}_n, \hat{H}_B + \epsilon_0 \hat{\tilde{a}}^\dagger_N \hat{\tilde{a}}_N] - \delta_{n1}\left(\frac{\kappa}{2}\hat{\tilde{p}}_n + e^{-A(N-1)}\sqrt{\kappa}\hat{P}^{(\text{in})}\right) \tag{25}$$

Crucially, we can immediately conclude that our chain is dynamically stable for any value of ϵ_0 and A: the spectrum is determined by the particle conserving Hamiltonian $\hat{H}_B + \epsilon_0 \hat{\tilde{a}}^\dagger_N \hat{\tilde{a}}_N$ and dissipation $\kappa/2$ on the first site.

Furthermore, we can readily use perturbation theory on Eqs.(24)-(25) to determine the effect of ϵ_0 on the eigenstates. As explained thoroughly in the main text, for $\epsilon_0 = 0$ they consist entirely of $\hat{\tilde{x}}$ or $\hat{\tilde{p}}$ excitations. For a small ϵ_0, the new eigenstates will have the form of a scattering state: it will consist of mostly $\hat{\tilde{x}}$ will a little bit of $\hat{\tilde{p}}$, while the converse is true of the formerly $\hat{\tilde{p}}$-only eigenstates. Moving back to the original unsqueezed frame (i.e. undoing Eqs.(22)-(23)), we see that the small change to the wavefunctions has been amplified/deamplified by an ammount which is exponentially large in system size.

These squeezing transformations however demonstrate taht we can go beyond perturbation theory and show that this amplification/deamplification persists to all orders in ϵ_0. Along with the previously-obtained quadrature-quadrature susceptibilities, the squeezing transformations gives

$$\chi^{xx}_{\epsilon_0}(n,m;t) = e^{A(n-m)}\,\text{Re}\,\tilde{\chi}_{\epsilon_0}(n,m;t) \tag{26}$$

$$\chi^{pp}_{\epsilon_0}(n,m;t) = e^{-A(n-m)}\,\text{Re}\,\tilde{\chi}_{\epsilon_0}(n,m;t) \tag{27}$$

$$\chi^{xp}_{\epsilon_0}(n,m;t) = -e^{-A(2N-n-m)}\,\text{Im}\,\tilde{\chi}_{\epsilon_0}(n,m;t) \tag{28}$$

$$\chi^{px}_{\epsilon_0}(n,m;t) = e^{A(2N-n-m)}\,\text{Im}\,\tilde{\chi}_{\epsilon_0}(n,m;t) \tag{29}$$

where $\tilde{\chi}_{\epsilon_0}(n,m;t)$ is the susceptibility matrix of the complex modes $\hat{\tilde{a}}_n$.

We already have the susceptibilities $\tilde{\chi}[n,m;\omega]$ of our tight-binding chain which incorporate the full effects of the the waveguide, which we derived in Methods. The frequency shift on the last site adds a term $-i\epsilon_0\delta_{n,N}\delta_{m,N}$ to the dynamical matrix. Dyson's equation in frequency space the gives:

$$\tilde{\chi}_{\epsilon_0}[n,m;\omega] = \tilde{\chi}[n,m;\omega] - i\epsilon_0\tilde{\chi}[n,N;\omega]\tilde{\chi}_{\epsilon_0}[N,m;\omega] \tag{30}$$

$$= \tilde{\chi}[n,m;\omega] - \frac{i\epsilon_0\tilde{\chi}[n,N;\omega]\tilde{\chi}[N,m;\omega]}{1 + i\epsilon_0\tilde{\chi}[N,N;\omega]}. \tag{31}$$

Since there is a driving force only on the first site, we just need to find the susceptibilities to a force on the first site:

$$\tilde{\chi}_{\epsilon_0}[n,1;\omega] = i^n \frac{U_{N-n}(\frac{\omega}{2J}) - \frac{\epsilon_0}{J}U_{N-1-n}(\frac{\omega}{2J})}{JU_N(\frac{\omega}{2J}) + (i\frac{\kappa}{2} - \epsilon_0)U_{N-1}(\frac{\omega}{2J}) - i\frac{\epsilon_0}{J}\frac{\kappa}{2}U_{N-2}(\frac{\omega}{2J})} \tag{32}$$

We now compute the steady state total photon number $\bar{n}_{\text{tot}}(\epsilon_0)$ when $\epsilon_0 \neq 0$. Recall we are interested in the regime where $\epsilon_0/\kappa \ll 1$ but $e^{A(N-1)}\epsilon_0/\kappa$ is not a priori small. The form of our susceptibilities Eqs.(26)-(29) implies that A doesn't effect

the spectrum, but just the residue of the poles as expected from our previous discussion. A non-zero value of ϵ_0 changes both the coherent drive-induced photon number, in addition to drive-independent photons generated from input vacuum fluctuations. The leading order correction to the total photon number when $\epsilon_0 \neq 0$ is therefore

$$\bar{n}_{\text{tot}}(\epsilon_0) = \bar{n}_{\text{tot}}(0) + (c\frac{\beta^2}{\kappa} + d)e^{4A(N-1)}(\frac{\epsilon_0}{\kappa})^2 + \mathcal{O}(e^{4A(N-2)}(\frac{\epsilon_0}{\kappa})^2) \tag{33}$$

where c and d are constants of order unity. Thus, using the definition of $Q(A, \epsilon_0)$ gives

$$Q(A, \epsilon_0) = \frac{4|\beta|^2 e^{2A(N-1)}/\kappa}{\bar{n}_{\text{tot}}(0) + \frac{1}{2}(c\frac{\beta^2}{\kappa} + d)e^{4A(N-1)}(\frac{\epsilon_0}{\kappa})^2 + \mathcal{O}(e^{4A(N-2)}(\frac{\epsilon_0}{\kappa})^2)}. \tag{34}$$

With the optimal amplification factor A^*

$$e^{4A^*(N-1)} = \frac{\kappa^2}{8\epsilon_0^2} \tag{35}$$

in conjunction with Eq. (6), we get

$$Q(A^*, \epsilon_0) = \left(\frac{1 - e^{-2A^*(N+1)}}{1 - e^{-4A^*}} + \mathcal{O}(e^{-2A^*(N-1)})\right)^{-1} \tag{36}$$

$$= 1 - \mathcal{O}((\frac{\epsilon_0^2}{\kappa^2})^{\frac{1}{N-1}}) \tag{37}$$

As in the main text. Note that we have taken the relevant limit $\beta^2/\kappa \gg 1$ such that we can ignore the amplified vacuum fluctuations to the total photon number

With the susceptibilities Eqs. (26)-29, we can also compute the quadrature-quadrature scattering matrix. If we first define

$$s[\omega] = 1 - \kappa\tilde{\chi}_{\epsilon_0}[1, 1; \omega] \tag{38}$$

$$= \frac{a[\omega] + ib[\omega]}{a[\omega] - ib[\omega]} \tag{39}$$

with

$$a[\omega] = JU_N(\frac{\omega}{2J}) - U_{N-1}(\frac{\omega}{2J})\epsilon_0 \tag{40}$$

$$b[\omega] = \frac{\kappa}{2}\left(\frac{\epsilon_0}{J}U_{N-2}(\frac{\omega}{2J}) - U_{N-1}(\frac{\omega}{2J})\right) \tag{41}$$

then using the input-output boundary conditions we find that the scattering matrix is

$$\boldsymbol{s}[\omega] = \begin{pmatrix} R[\omega] & -T[\omega]e^{-2A(N-1)} \\ T[\omega]e^{2A(N-1)} & R[\omega] \end{pmatrix} \tag{42}$$

with

$$R[\omega] = \frac{1}{2}\left(s[\omega] + s^*[-\omega]\right) \tag{43}$$

$$T[\omega] = \frac{1}{2i}\left(s[\omega] - s^*[-\omega]\right). \tag{44}$$

Note that $|s[\omega]|^2 = 1$, which implies $|R[\omega]|^2 + |T[\omega]|^2 = 1$. The zero-frequency component of $R(\epsilon_0)$ and $T(\epsilon_0)$ are then:

$$R(\epsilon_0) = -\frac{(\frac{\kappa}{2})^2 - \epsilon_0^2}{(\frac{\kappa}{2})^2 + \epsilon_0^2} \tag{45}$$

$$T(\epsilon_0) = \frac{\kappa\epsilon_0}{(\frac{\kappa}{2})^2 + \epsilon_0^2} \tag{46}$$

as in the main text.
